# A Rare Case of Melanosis of the Hard Palate Mucosa in a Patient with Chronic Myeloid Leukemia

**DOI:** 10.1155/2015/817094

**Published:** 2015-09-15

**Authors:** Umberto Romeo, Gaspare Palaia, Paolo Junior Fantozzi, Gianluca Tenore, Daniela Bosco

**Affiliations:** ^1^Department of Oral and Maxillofacial Sciences, “Sapienza” University of Rome, Via Caserta 6, 00161 Rome, Italy; ^2^Department of Radiological, Oncological and Anatomo-Pathological Sciences, “Sapienza” University of Rome, Viale Regina Elena 324, 00161 Rome, Italy

## Abstract

Imatinib Mesylate, also known as Gleevec or ST1-571, is a tyrosine-kinase inhibitor used as the gold standard medication for the chronic myeloid leukemia (CML); Imatinib has indeed deeply revolutionized the CML therapy allowing most patients to have a good quality of life. Despite its beneficial effects, Imatinib has significant side effects such as mucosal pigmentation. A 72-year-old female having an Imatinib induced mucosal pigmentation is presented: she has been treated with Imatinib since 2003 and only in 2014 discovered, during a routine dental visit, having a pigmented lesion on her hard palate mucosa. Histopathologically, the lesion shows the deposition of fine dark brown spherical bodies within the lamina propria and cloaked in between the collagen fibers. There was no sign of inflammation, hyperplasia, or hemorrhage in the tissue.

## 1. Introduction

Pigmented lesions of the oral cavity are common oral lesions that may present as a solitary lesion, as a manifestation of systemic pathology, or as a collateral effect of drug therapy [1]. The etiology of oral pigmentation may range from simple benign lesions, such as a blue nevus, to complex and malignant disorders, such as oral melanoma. The differential diagnosis includes physiological pigmentation such as those typically seen in African Americans, postinflammatory melanosis, amalgam tattoo, smoking related pigmentation, blue nevus, and malignant melanoma. Peutz-Jeghers syndrome, Addison disease, and some other rare diseases, such as polyostotic dysplasia, hyperthyroidism, and Nelson syndrome, could be also associated with an oral pigmented lesion.

Moreover, a number of drugs, such as antimalarials, tetracyclines, and antiretroviral and chemotherapeutic drugs, may also cause oral pigmentations. One of these chemotherapeutic drugs, Imatinib Mesylate, also known as Gleevec or ST1-571, is a tyrosine-kinase inhibitor that targets Bcr-Abl protein, c-Kit, and platelet derived growth factor receptor and is used as a first-line treatment for chronic myeloid leukemia (CML) and gastrointestinal stromal tumor (GIST) [2, 3]. Chronic myeloid leukemia is a slow-growing tumor of white blood cells, characterized by an unregulated growth of the myeloid precursor cells and its accumulation both in the bone marrow and the lymphoid organs. Chronic myeloid leukemia is more common in males than females and appears more in patients between 25 and 60 years. The cause of CML is the translocation of regions of the BCR and ABL genes to form a BCR-ABL fusion gene. In at least 90 percent of cases, this event is a reciprocal translocation termed t(9;22), which forms the Philadelphia (Ph) chromosome. The product of the BCR-ABL gene, the BCR-ABL protein, is a constitutively active protein tyrosine kinase with an important role in the regulation of cell growth [4]; its presence is a strong indicator for CML (since 95% of people with CML have it) but not sufficient on its own for a diagnosis, because 30% of people with ALL will also show Ph+ in their genome. The 9;22 translocation leads to a chimeric gene fusion through the bonding of Abl-1 (Abelson) gene located on chromosome 9, with a part of the Bcr (breakpoint cluster region) on a truncated chromosome 22. This way, BCR-ABL acts as an oncogene that overexpresses a tyrosine-kinase protein that stimulates the leukemic growth of myeloblasts [5]. Chronic myeloid leukemia has been treated for years with both hydroxyurea and bone marrow transplant, which, until the discovery of Imatinib, has been the only successful therapy in 70% of the cases.

With the introduction of Imatinib Mesylate (ST1-571 or Gleevec) the therapeutic options have significantly improved. This treatment modality allows most patients to have a good quality of life when compared to the other chemotherapy drugs [6–8]. Despite its enormous therapeutic effects, Gleevec has very common adverse effects including nausea, fluid retention, lowered resistance to infection (due to neutropenia), and, sometimes, congestive cardiac failure. Dermatological side effects have also been very common such as rash, superficial edema, GVH-like disease, erythroderma, and lichenoid eruptions. On the other hand, intraoral side effects seem to be very rare, such as lichenoid reactions and dental and oral mucosal pigmentation [9].

Herein we report a case of oral pigmentation involving the hard palate mucosa in a patient on Imatinib therapy.

## 2. Case Report

**Figure 1:**
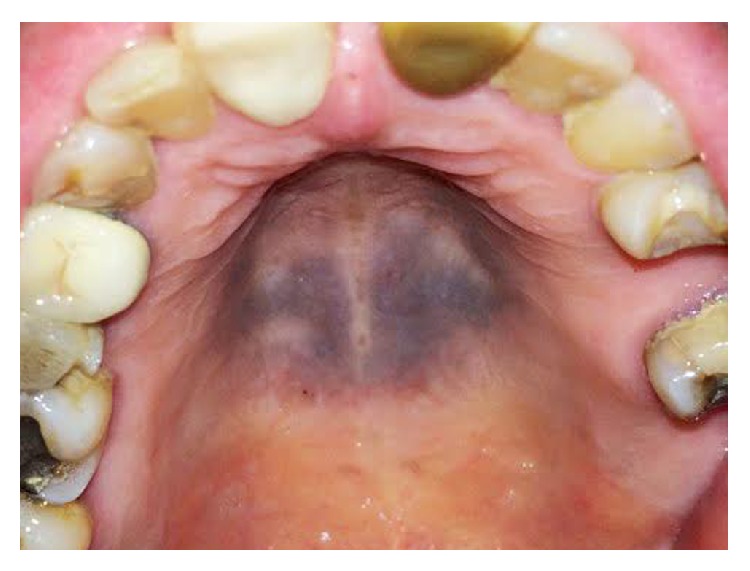
Diffuse, blue-grey pigmented lesion of the hard palate mucosa of our case.

A 72-year-old Spanish woman was referred in October 2014 from her regular dentist to the Unit of Oral Medicine, Sapienza University of Rome, for evaluation of a pigmented lesion on the hard palate mucosa. The lesion, discovered during a routine dental examination, appeared as a single, flat, painless lesion, with a blue-grey color, blurred edges, and was located in the center of the hard palate mucosa. There were areas of physiologic mucosa above the palatal raphe (Figure 1). The patient was unaware of the lesion, and she stated that she had no history of trauma to the hard palate.

Her medical history was significant for gastroesophageal reflux disease (GERD), diuresis, and CML. She is being treated with Imatinib Mesilate 400 mg per day since 2003 for CML. She is also taking medications for GERD (esomeprazole) and for diuresis (amiloride HCl-hydrochlorothiazide).

The patient stated she has never taken minocycline, hydroxyurea, and/or antimalarial drugs. Furthermore she reports she has never smoked or drank alcohol and has never been in contact with heavy metals. During the visit we did not find any other pigmented lesion on the thorax/back, skin, or the fingernails. An incisional biopsy of the hard palate mucosa was scheduled and performed two weeks later. The biopsy was executed by taking a square section of the right hard palate mucosa representative of the lesion and fixed in 4% buffered formaldehyde. It was 10 × 4 mm with a depth measuring 2–4 mm.

## 3. Histopathological Findings

**Figure 2:**
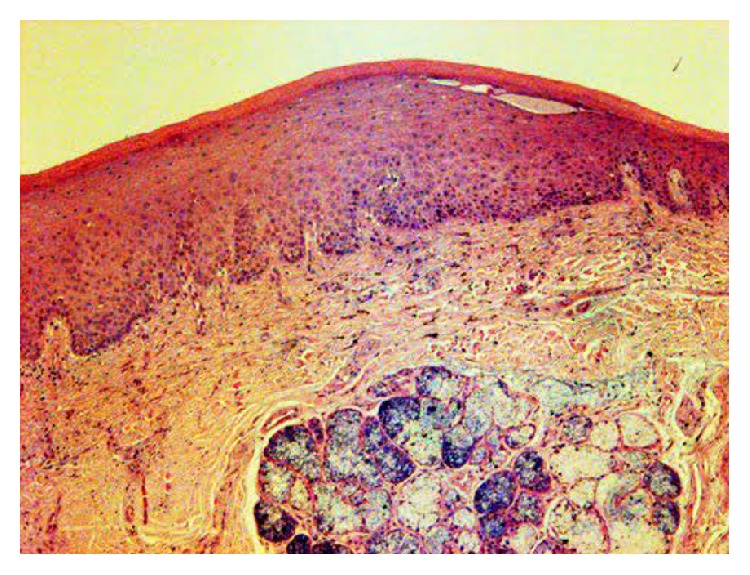
Palatal mucosa with a minor salivary gland containing pigment within the lamina propria (HE ×40).

**Figure 3:**
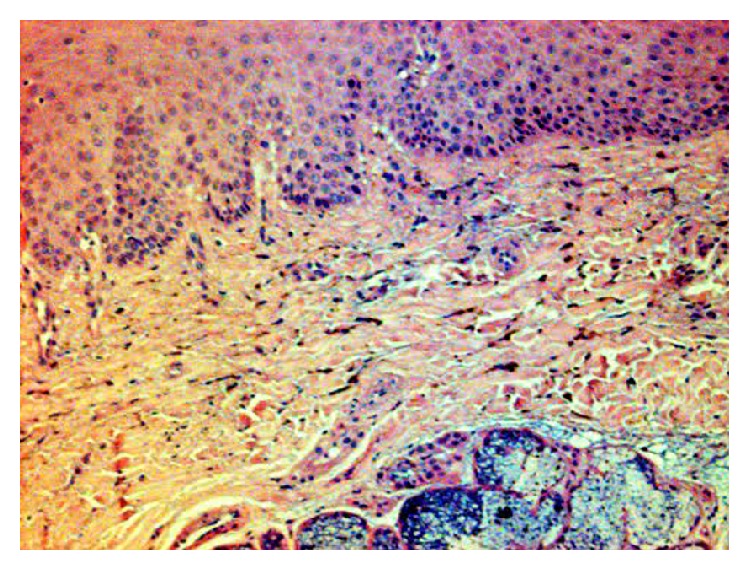
Fine spherical bodies lying in the lamina propria; absence of hyperplasia or inflammation or hemorrhage (HE ×200).

**Figure 4:**
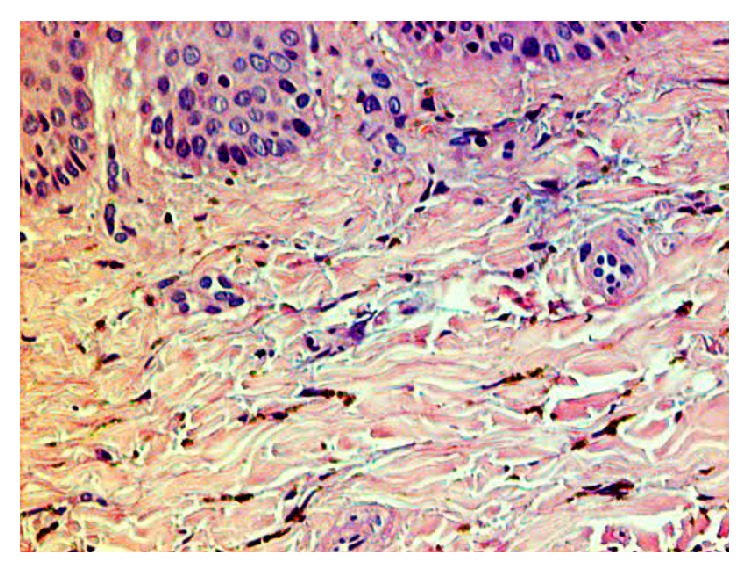
Fine spherical brown particles cloaked in between the collagen fibers in the lamina propria (HE ×400).

Standard hematoxylin and eosin stained sections were performed; the microscopic examination of the tissue showed a squamous tissue and an underlying tissue containing a minor salivary gland (Figure 2). There were no signs of cellular atypia, melanocytic hyperplasia, hemorrhagic circumstances, and inflammation. Hard palate mucosa showed the presence of minimal brown spherical bodies located within the lamina propria, lying in between the collagen fibers (Figures 3 and 4).

Established on clinical and histological data, the diagnosis was mucosal pigmentation related to Imatinib Mesylate therapy.

## 4. Discussion

The above patient after long-term use (11 years) of Imatinib Mesylate developed a diffuse blue-grey pigmentation of the hard palate mucosa. The lesion, histopathologically, showed the presence of fine brown spherical particles within the lamina propria, which were both positive for Prussian blue and Fontana-Masson stains, indicating the presence of hemosiderin and melanin, respectively. There was no evidence of malignancy, and both the clinical and histopathological presentation confirmed the diagnosis of drug-related melanosis of the mucosa, Imatinib in this case.

Imatinib Mesylate works by inhibiting BCR-ABL by binding the ATP site in an “off mode” so that the enzymatic activity of the protein is not catalyzed anymore [7, 8].

Dermatological side effects of Imatinib are common such as rash, superficial edema, Graft Versus Host-like disease, erythroderma, and lichenoid eruptions. Despite these common side effects, Gleevec has rarely been reported to produce diffuse hyperpigmentation of the palatal mucosa.

Generally, medication-induced melanosis in the oral mucosa can be attributed to any one of the following mechanisms: pigmented breakdown of drug products, drugs inducing melanin formation, or drug metabolites chelated with iron and melanin.

The pathogenesis of oral mucosal pigmentation in patients taking Imatinib still remains unclear; it has been suggested that Imatinib blocks the binding on the c-kit receptors (a growth factor receptor found on skin cell, melanocytes, and mast cells) and subsequently alters both melanogenesis and melanocyte homeostasis [2, 9]. However, pigmented depositions in the oral mucosa indicate much different pathologies and are challenging to diagnose correctly. Differential diagnosis includes physiological pigmentation, amalgam tattoo, melanotic macule, smoker's melanosis, melanocytic nevus, malignant melanoma, and drug-induced melanosis.

The two most common types of melanotic alteration are physiological pigmentations, which are related to racial melanosis (mostly diffuse changes on the attached gingiva) and postinflammation melanosis, as a consequence of a trauma.

Melanotic macule and melanocytic nevus are rare, but in 90% of cases they are located in the hard palate mucosa, which is why they were included in the differential diagnosis.

Pigmented lesions related to systemic diseases were also considered. Addison's disease and Peutz-Jeghers syndrome could lead to an oral melanosis condition such as diffuse oral macules and oral ephelides [2–4, 10, 11]. In regard to medication-related melanosis other than Imatinib-antimalarials, minocycline, anti-inflammatory drugs for treating leprosy, and conjugated estrogens are considered.

The reason why the melanin and iron accumulate in this part of the oral cavity is still unknown; what is known is that they accumulate due to drug metabolites that chelate them, just like minocycline and antimalarial drugs do.

In summary we present a case of melanosis of the hard palate mucosa related to Imatinib; the lesion is benign and therefore no treatment is needed [2, 10, 11].
